# High intraoperative fluid load associated with prolonged length of hospital stay and complications after non-cardiac surgery in neonates

**DOI:** 10.1007/s00431-024-05628-x

**Published:** 2024-06-10

**Authors:** Minyue Qian, Jialian Zhao, Kai Zhang, Wenyuan Zhang, Chunyi Jin, Binbin Cai, Zhongteng Lu, Yaoqin Hu, Jinjin Huang, Daqing Ma, Xiangming Fang, Yue Jin

**Affiliations:** 1 Department of Anesthesiology, Children’s Hospital, Zhejiang University School of Medicine, National Clinical Research Center for Child Health, Hangzhou, 310052 China; 2 Department of Anesthesiology and Intensive Care, The First Affiliated Hospital, Zhejiang University School of Medicine, Hangzhou, Zhejiang 310003 China; 3 Perioperative and Systems Medicine Laboratory, Children’s Hospital, Zhejiang University School of Medicine, National Clinical Research Center for Child Health, Hangzhou, China; 4 Division of Anaesthetics, Pain Medicine and Intensive Care, Department of Surgery and Cancer, Faculty of Medicine, Imperial College London, Chelsea and Westminster Hospital, London, UK

**Keywords:** Neonate, Non-cardiac surgery, Intraoperative fluid management, Outcome, Mortality, Length of stay, Postoperative complications

## Abstract

Inappropriate perioperative fluid load can lead to postoperative complications and death. This retrospective study was designed to investigate the association between intraoperative fluid load and outcomes in neonates undergoing non-cardiac surgery. From April 2020 to September 2022, 940 neonates who underwent non-cardiac surgery were retrospectively enrolled and their perioperative data were harvested for further analysis. According to recorded intraoperative fluid volumes defined as ml.kg^−1^ h^−1^, patients were mandatorily divided into quintile with fluid load as restrictive (quintile 1, Q1), moderately restrictive (Q2), moderate (Q3), moderately liberal (Q4), and liberal (Q5). The primary outcomes were defined as prolonged length of hospital stay (LOS) (postoperative LOS ≥ 14 days), complications beyond prolonged LOS, and 30-day mortality. Secondary outcomes included postoperative complications within 14 days of hospital stay. The intraoperative fluid load was in Q1 of 6.5 (5.3–7.3) (median and IQR); Q2: 9.2 (8.7–9.9); Q3: 12.2 (11.4–13.2); Q4: 16.5 (15.4–18.0); and Q5: 26.5 (22.3–32.2) ml.kg^−1^ h^−1^. The odd of prolonged LOS was positively correlated with an increase fluid volume (Q5 quintile: OR 2.602 [95% CI 1.444–4.690], *P* = 0.001), as well as complications beyond prolonged LOS (Q5: OR 3.322 [95% CI 1.656–6.275], *P* = 0.001). The overall 30-day mortality rate was increased with high intraoperative fluid load but did not reach to a statistical significance after adjusted with confounders. Furthermore, the highest quintile of fluid load (26.5 ml.kg^−1^ h^−1^, IQR [22.3–32.2]) (Q5 quintile) was significantly associated with longer postoperative mechanical ventilation time compared with Q1 (Q5: OR 2.212 [95% CI 1.101–4.445], *P* = 0.026).

*    Conclusion*: Restrictive intraoperative fluid load had overall better outcomes, whilst high fluid load was significantly associated with prolonged LOS and complications after non-cardiac surgery in neonates.

*    Trial registration*: Chictr.org.cn Identifier: ChiCTR2200066823 (December 19, 2022).

**Table:** 

**What is Known:** *• Inappropriate perioperative fluid load can lead to postoperative complications and even death.*	
**What is New:** *• High perioperative fluid load was significantly associated with an increased length of stay after non-cardiac surgery in neonates, whilst low fluid load was consistently related to better postoperative outcomes.*

**What is Known:**

*• Inappropriate perioperative fluid load can lead to postoperative complications and even death.*

**What is New:**

*• High perioperative fluid load was significantly associated with an increased length of stay after non-cardiac surgery in neonates, whilst low fluid load was consistently related to better postoperative outcomes.*

## Introduction

Perioperative fluid management is recognized as a crucial factor affecting surgical outcomes [1, 2]. Proper administration of intravenous fluids is essential for maintaining normal physiological function in pediatric surgical patients. However, low blood volume during perioperative period can result in decreased cardiac output and reduced tissue perfusion, potentially leading to acute kidney injury. Conversely, high blood volume may be linked to an increased risk of adverse events, including tissue edema, acid-base disorders, heart failure, and even death [3, 4].

These postoperative complications increased postoperative mortality, prolonged length of hospital stay (LOS), and re-admitted to hospital [5], thereby negatively affected long-term survival [2, 6, 7]. The choice between restrictive and liberal fluid therapy is still debating. Currently, “goal-directed fluid therapy” (GDFT) is steadily gaining popularity for appropriate perioperative fluid management, as it has been shown to reduce perioperative complications and mortality [8–10]. A study by Osawa and colleagues demonstrated that perioperative GDFT reduced the incidence of low cardiac output syndrome in patients undergoing cardiac surgery [11]. However, GDFT has not yet been optimized for clinical use in neonates. Therefore, it is crucial to determine the optimal intravenous infusion volume to enhance perioperative fluid management in neonates, aiming to maximize therapeutic benefits while minimizing iatrogenic toxicity [12].

In the past decades, perioperative fluid management has been focused on adults and children but studies on neonates are limited [1, 8, 13]. In this study, we investigated a large cohort of neonates undergoing non-cardiac surgery and analyzed the impact of high or low intraoperative fluid load on postoperative clinical outcomes aiming to provide guidance on intraoperative fluid management for neonates.

## Methods

### Study design and setting

**Fig. 1:**
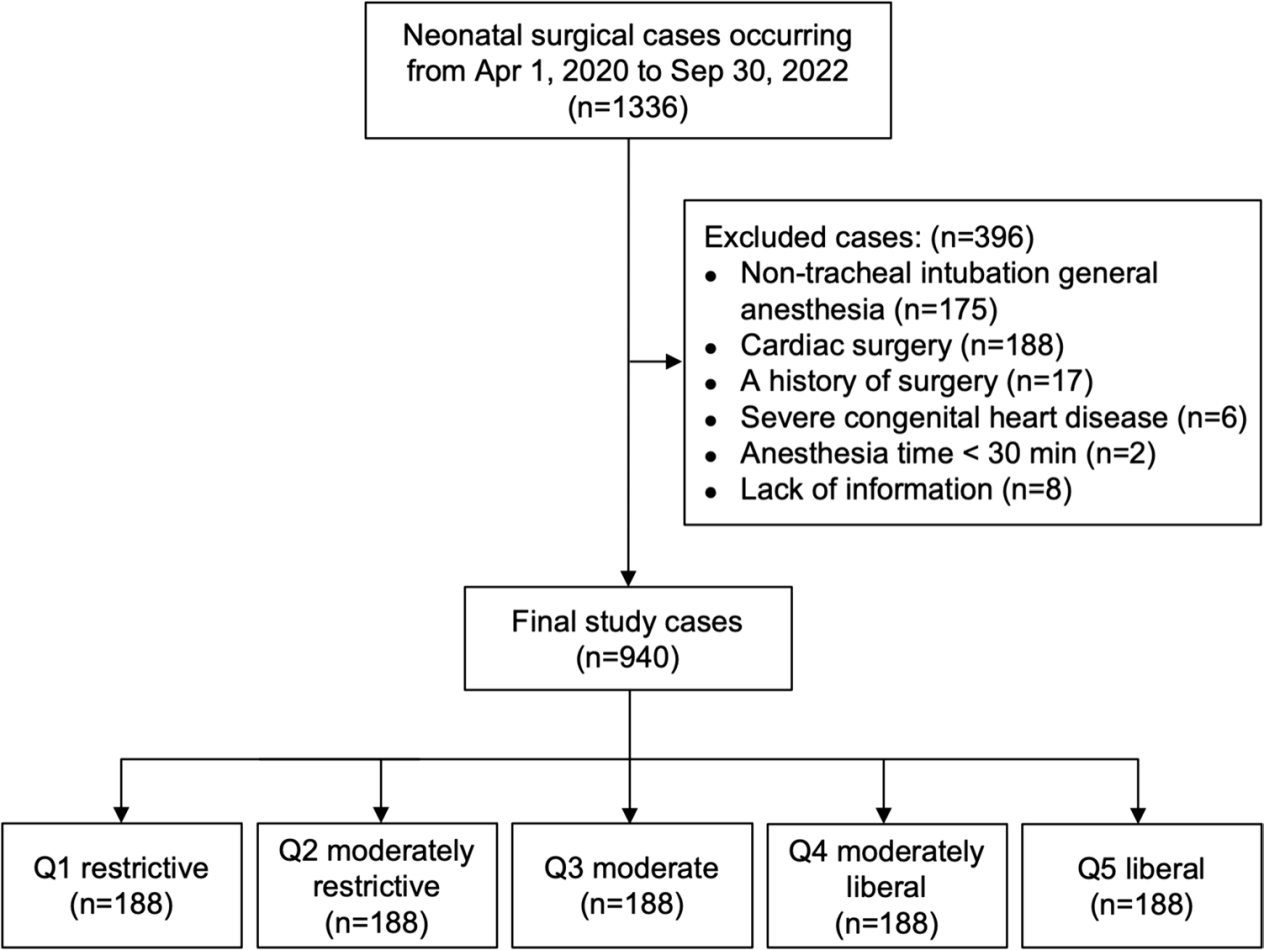
Patients’ categorized flow diagram according to intraoperative fluid load in this group patient population

The study was approved by the Ethics Committee Board of Children’s Hospital, Zhejiang University School of Medicine (2022-IRB-273, December 6, 2022), registered at chictr.org.cn. (ChiCTR2200066823, December 19, 2022) and conducted a retrospective analysis of surgical data from April 2020 to September 2022 in the same hospital as above. All neonates who underwent non-cardiac surgery and general anesthesia with intraoperative endotracheal intubation were included in the study (Fig. 1). Demographics, intraoperative, and outcome data were collected from clinical and administrative database. The excluded criteria were as follows: (1) non-tracheal intubation general anesthesia, (2) undergoing cardiac surgery, (3) a history of surgery, (4) severe congenital heart disease, (5) anesthesia time less than 30 min, and (6) data incompletion.

### Exposure variable

The total fluid load was calculated based on the anesthesia record and defined as the sum effective volumes of crystalloid, colloid, and blood products administered between onset of anesthesia till surgery completion before arrived to the post anesthesia care unit (PACU) or intensive care unit (ICU) [1, 14]. Patients were categorized into five quintiles (Q) based on the fluid volume per kilogram of body weight per hour during intraoperative period: restrictive (quintile 1, Q1), moderately restrictive (quintile 2, Q2), moderate (quintile 3, Q3), moderately liberal (quintile 4, Q4), and liberal (quintile 5, Q5).

### Outcome measures

The primary outcomes included a prolonged LOS (postoperative hospital stay equal and more than 14 days) [15, 16], complications beyond prolonged LOS, and 30-day mortality after surgery. The secondary outcomes were postoperative complications including (i) postoperative pulmonary complications (PPCs), (ii) acute kidney injury (AKI), (iii) hepatic dysfunction, (iv) surgical site infection, (v) thrombus formation, and (vi) postoperative hypotension required vasopressors. The data of postoperative mechanical ventilation time equal and more than 24 h and postoperative blood transfusion were also collected.

Postoperative pulmonary complications were defined as the occurrence of respiratory infection, respiratory failure, pleural effusion, atelectasis, pneumothorax, bronchospasm, or aspiration pneumonitis [17]. AKI was defined as an increase by 0.3 mg/dL or by 50% relative to its before of serum creatinine compared to preoperative levels within 48 h after surgery, or the presence of an AKI diagnostic code within 7 days after surgery [18, 19]. Postoperative hypotension, in preterm neonates, was defined as a mean blood pressure below 30 mmHg [20, 21], whilst, for term neonates, it is lower than 39 mmHg or decreased by 20% relative to the baseline [22].

### Statistical analysis

Quantitative data were expressed as median and interquartile ranges (IQRs) as all data are not normally distributed. They were analyzed with the Kruskal-Wallis test. Categorical data were reported as patients’ numbers and percentages (*n*, %) and compared with chi-square test or fisher’s exact test. Binomial logistic regressions were employed to assess the association between fluid volume quintiles and the binary outcomes. Adjusted odds ratios (OR) and 95% confidence intervals (CI) were calculated to determine the associations between fluid quintiles and outcomes. The covariates included in the models were age, weight, premature, gender, ASA physical status, admission type, surgery type, anesthesia duration, intraoperative lactic acid, intraoperative blood loss, and intraoperative vasopressor drugs. Survival analysis was conducted using Kaplan-Meier curves for each intervention group, and the Log Rank test was used for comparison. A double-tailed *P* < 0.05 was considered to be statistically significant. All statistical analyses were performed using SPSS 24.0 (IBM, USA) or GraphPad Prism 8 (GraphPad Software, USA).

## Results

### Study cohort and patient characteristics

A total of 940 neonates met the study criteria in the period from April 2020 to September 2022 (Fig. 1). The cohort had a median age (IQR) of 6.0 (2.0–14.0) days and the bodyweight of 2.9 (2.3–3.4) kg. Among the cohort, 521 (55.4%) were males. The majority of patients had ASA status Ι and II (787, 83.7%). The most common surgery type was gastrointestinal (758, 80.6%), followed by neurosurgery (91, 9.7%), and thoracic (61, 6.5%). The median (IQR) surgery time and anesthesia time were 73.0 (44.0–102.0) and 127.0 (90.0–160.0) min, respectively (Table 1).

**Table 1:** Summary of patient characteristics

	Overall cohort (*n* = 940)	Quintile of fluid load					
		Q1 restrictive (*n* = 188)	Q2 moderately restrictive (*n* = 188)	Q3 moderate (*n* = 188)	Q4 moderately liberal (*n* = 188)	Q5 liberal (*n* = 188)	*P*
Age (day)	6.0 (2.0–14.0)	7.0 (3.0–16.0)	6.0 (3.0–15.0)	5.0 (2.0–12.0)	5.0 (2.0-12.8)	5.0 (2.0–13.0)	0.16
Weight (kg)	2.9 (2.3–3.4)	3.3 (2.8–3.7)	3.2 (2.7–3.5)	3.0 (2.5–3.4)	2.8 (2.1–3.2)	2.1 (1.6–2.8)	< 0.001
Premature	337 (35.9)	34 (18.1)	42 (22.3)	54 (28.7)	72 (38.3)	135 (71.8)	< 0.001
CAG (week)	39.0 (36.0–41.0)	40.0 (39.0–41.0)	40.0 (39.0–41.0)	39.0 (38.0–41.0)	39.0 (35.3–40.0)	35.5 (32.0–38.0)	< 0.001
Gender							0.50
Male	521 (55.4)	109 (58.0)	112 (59.6)	100 (53.2)	103 (54.8)	97 (51.6)	
Female	419 (44.6)	79 (42.0)	76 (40.4)	88 (46.8)	85 (45.2)	91 (48.4)	
ASA physical status							< 0.001
Ι	82 (8.7)	32 (17.0)	22 (11.7)	16 (8.5)	10 (5.3)	2 (1.1)	
ΙΙ	705 (75.0)	143 (76.1)	152 (80.9)	150 (79.8)	144 (76.6)	116 (61.7)	
ΙΙΙ	137 (14.6)	13 (6.9)	14 (7.4)	21 (11.2)	31 (16.5)	58 (30.9)	
IV	15 (1.6)	0	0	1 (0.5)	3 (1.6)	11 (5.9)	
V	1 (0.1)	0	0	0	0	1 (0.5)	
Admission type							< 0.001
Emergency	737 (78.4)	142 (75.5)	137 (72.9)	141 (75.0)	155 (82.4)	162 (86.2)	
Elective	203 (21.6)	46 (24.5)	51 (27.1)	47 (25.0)	33 (17.6)	26 (13.8)	
Surgery type							< 0.001
Gastrointestinal	758 (80.6)	132 (70.2)	144 (76.6)	150 (79.8)	160 (85.1)	172 (91.5)	
Neurosurgery	91 (9.7)	26 (13.8)	22 (11.7)	19 (10.1)	16 (8.5)	8 (4.3)	
Thoracic	61 (6.5)	14 (7.4)	12 (6.4)	17 (9.0)	11 (5.9)	7 (3.7)	
ENT and ophthalmic	12 (1.3)	2 (1.1)	8 (4.3)	0	1 (0.5)	1 (0.5)	
Urological	18 (1.9)	14 (7.4)	2 (1.1)	2 (1.1)	0	0	
Surgery duration (min)	73.0 (44.0–102.0)	53.5 (32.3–83.5)	62.5 (38.3–90)	69.5 (43.0–100.0)	85.0 (50.0–117.5)	87.0 (67.0–119.0)	< 0.001
Anesthesia duration (min)	127.0 (90.0–160.0)	110.0 (79.3–137.8)	116.0 (82.0–148.0)	125.0 (87.5–162.0)	137.0 (96.5–176.8)	143.0 (122.0–170.0)	< 0.001
Intraoperative lactic acid	1.5 (1.0–2.4)	1.3 (1.0–2.2)	1.3 (1.0–1.8)	1.4 (1.1–2.2)	1.4 (1.0–2.4)	2.0 (1.3–3.3)	< 0.001
Intraoperative blood loss (ml)	2.0 (1.0–5.0)	2.0 (1.0–2.0)	2.0 (1.0–5.0)	2.0 (1.0–5.0)	2.0 (2.0–5.0)	5.0 (2.0–10.0)	< 0.001
Intraoperative vasopressor drugs	201 (21.4)	14 (7.4)	18 (9.6)	24 (12.8)	53 (28.2)	92 (48.9)	< 0.001
Fluid load (ml kg^−1^ h^−1^)	12.2 (8.7–18.0)	6.5 (5.3–7.3)	9.2 (8.7–9.9)	12.2 (11.4–13.2)	16.5 (15.4–18.0)	26.5 (22.3–32.2)	< 0.001

Data are presented as median (quartile) or *n* (%)

*ASA* American Society of Anesthesiologists, *CAG* corrected gestational age, *ENT* ear-nose-throat

During surgery, the median (IQR) fluid load received was 12.2 (8.7–18.0) ml kg^−1^ h^−1^ (Table 1). The fluid load received in each of the five quintiles was as follows: Q1: lowest (*n* = 188): 6.5 (5.3–7.3) ml kg^−1^ h^−1^; Q2: low (*n* = 188): 9.2 (8.7–9.9); Q3: moderate (*n* = 188): 12.2 (11.4–13.2); Q4: high (*n* = 188): 16.5 (15.4–18.0); and Q5: highest (*n* = 188): 26.5 (22.3–32.2).

### Primary outcome

**Fig. 2:**
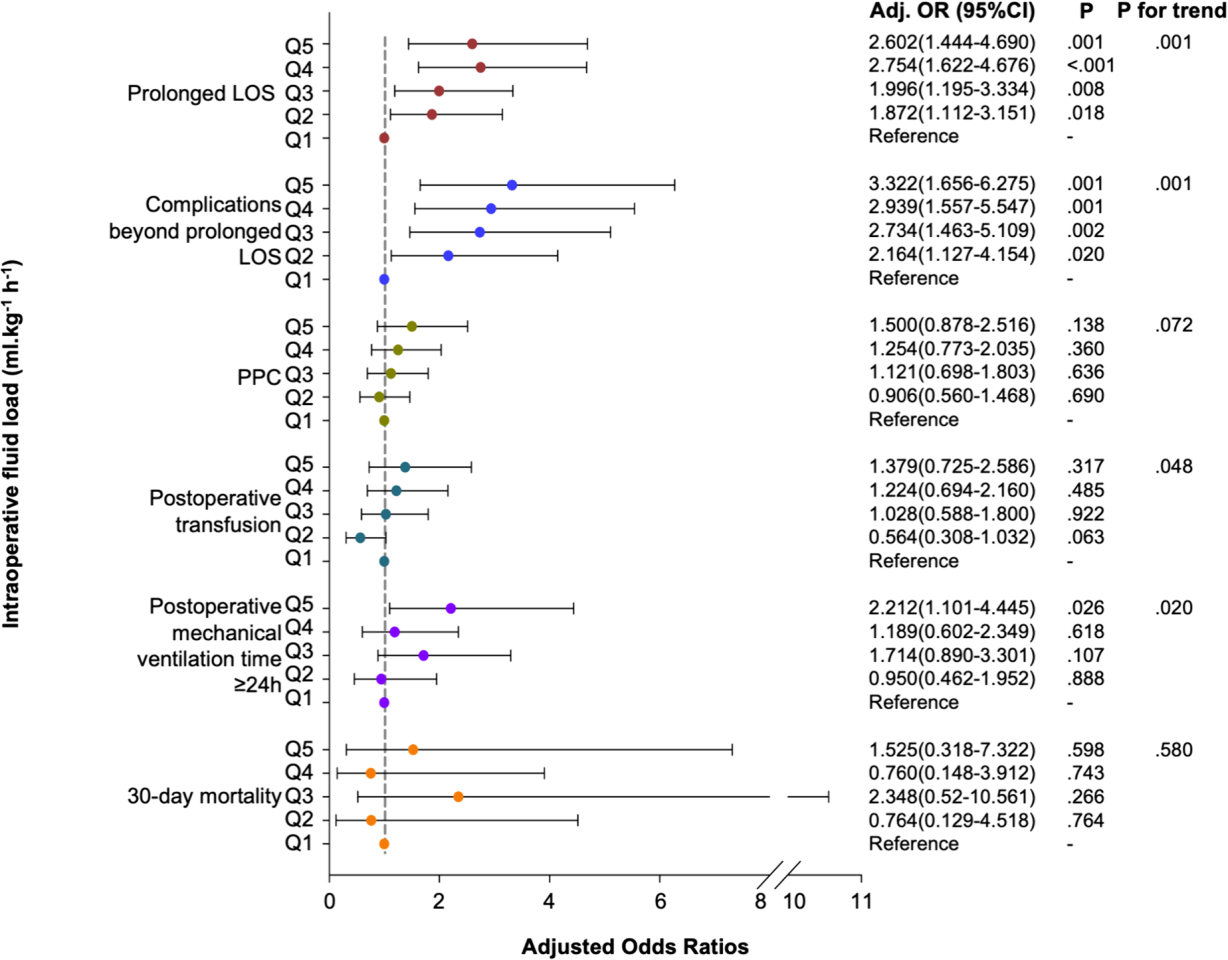
Effect of intraoperative fluid load on postoperative outcomes. The association between total intraoperative fluid load and postoperative outcomes was analyzed using multivariable logistic regression. The association between intraoperative fluid load and prolonged LOS, complications with prolonged LOS, PPCs, postoperative transfusion, postoperative mechanical ventilation time ≥ 24 h, and 30-day mortality were analyzed using multivariable models. The adjusted OR with 95% CI for all outcomes during the study period is shown (compared to Q1). CI: confidence intervals; OR: odds ratios; LOS: length of stay; PPCs: postoperative pulmonary complications

**Fig. 3:**
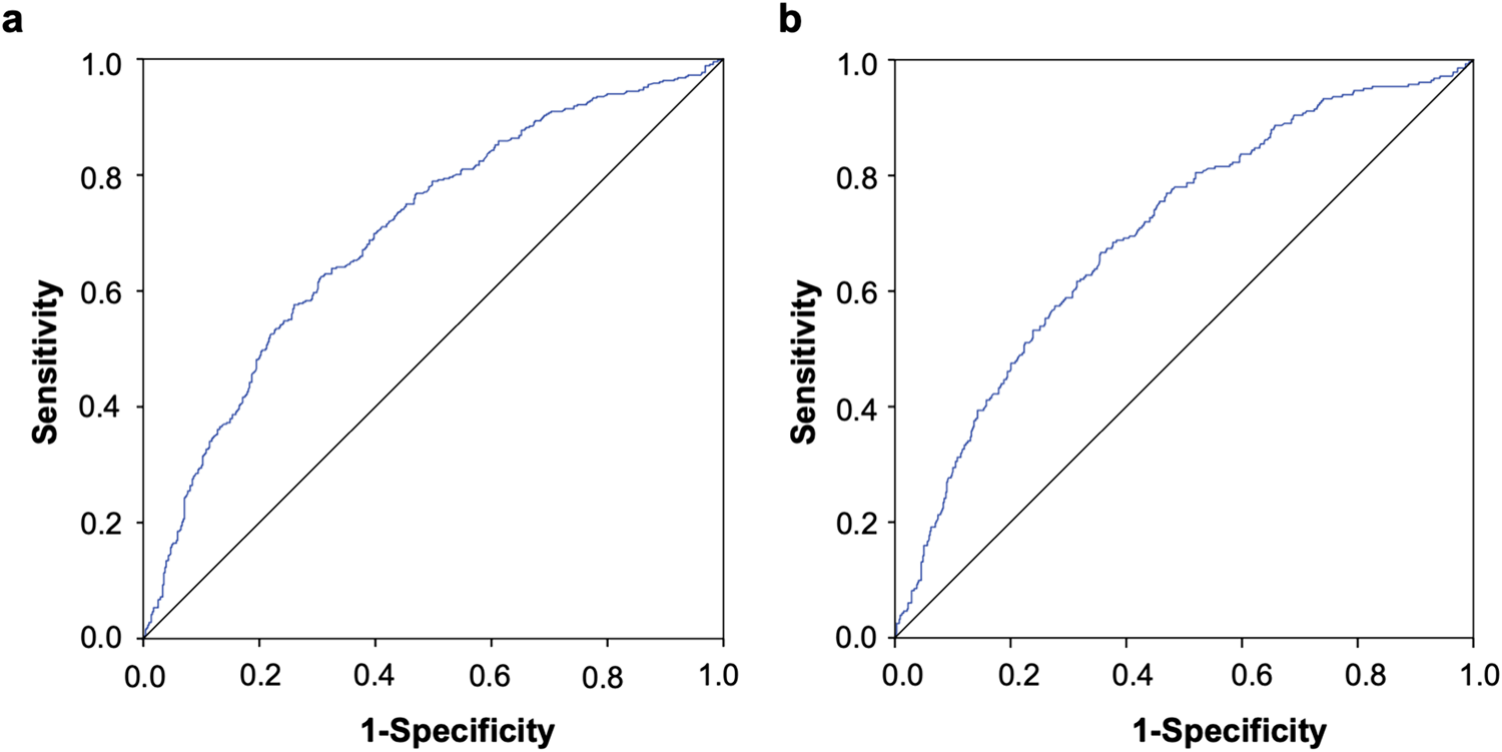
Receiver operating characteristic (ROC) curves drawn for the model. **a** Prolonged LOS, the area under the ROC curve (AUC) = 0.701 (95% CI: 0.668–0.734). **b** Complications beyond prolonged LOS, the area under the ROC curve (AUC) = 0.698 (95% CI: 0.662–0.735)

Of the total patients, 432 (46.0%) had a prolonged LOS. Adjusted outcomes revealed that increasing fluid volumes were significantly associated with prolonged LOS (Q5: OR 2.602 [95% CI 1.444–4.690], Q4: OR 2.754 [95% CI 1.622–4.676], Q3: OR 1.996 [95% CI 1.195–3.334], Q2: OR 1.872 [95% CI 1.112–3.151], all *P* < 0.05, *p* for trend = 0.001) (Fig. 2). The area under the ROC curve (AUC) was 0.701 (95% CI: 0.668–0.734) with a cutoff value of 13.15 ml kg^−1^ h^−1^ (Fig. 3a).

Similar results were found for complications beyond prolonged LOS (282, 30.0%). Increase liquid volumes were consistently associated with a higher risk of prolonged LOS (all *P* < 0.05, *P* for trend = 0.001) (Fig. 2). The area under the ROC curve (AUC) was 0.698 (95% CI: 0.662–0.735) with a cutoff value of 13.20 ml kg^−1^ h^−1^) (Fig. 3b).

**Fig. 4:**
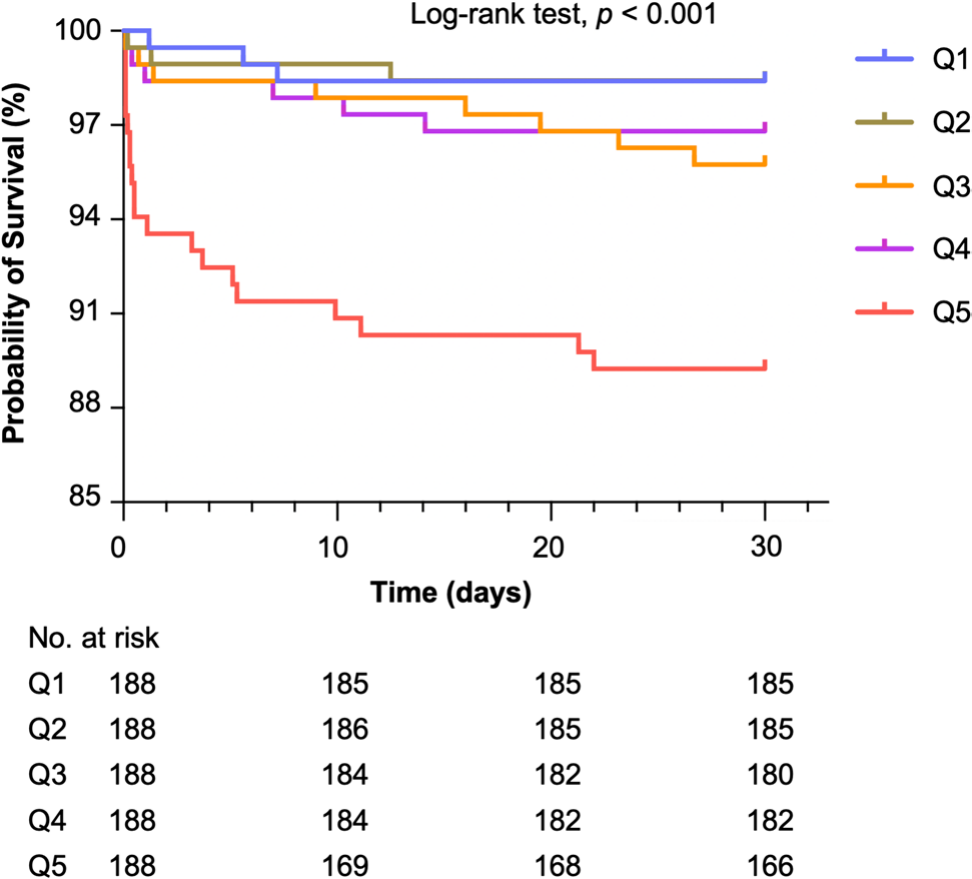
Survival curve of the 30-day mortality after non-cardiac surgery in different groups

Among the patients, 42 (4.5%) died within 30-days after surgery (Table 2). The 30-day mortality in different groups is shown in Fig. 4. There was a statistical difference in survival rates among the five groups (*P* < 0.001) (Fig. 4). However, high intraoperative fluid load was not found to be associated with 30-day mortality after adjusted with confounders (Fig. 2).

**Table 2:** Effect of intraoperative fluid load on postoperative outcomes

	Overall cohort (*n* = 940)	Quintile of fluid load					
		Q1 restrictive (*n* = 188)	Q2 moderately restrictive (*n* = 188)	Q3 moderate (*n* = 188)	Q4 moderately liberal (*n* = 188)	Q5 liberal (*n* = 188)	*P*
Postoperative LOS, days	12.8 (7.3–25.4)	8.2 (5.5–13.3)	10.6 (6.6–16.8)	12.4 (7.4–23.0)	16.9 (9.1–32.3)	25.2 (12.9–51.6)	< 0.001
30-day mortality	42 (4.5)	3 (1.6)	3 (1.6)	8 (4.3)	6 (3.2)	22 (11.7)	< 0.001
ICU admission	871 (92.7)	157 (83.5)	164 (87.2)	178 (94.7)	184 (97.9)	188 (100.0)	< 0.001
Postoperative ICU stay, days	1.7 (0.8–8.7)	0.9 (0.7–2.9)	1.1 (0.8–4.8)	1.6 (0.8-8.0)	1.7 (0.8–9.6)	5.7 (1.7–37.8)	< 0.001
Postoperative mechanical ventilation time, hours	14.3 (7.7–26.6)	10.2 (6.6–18.1)	10.5 (6.6–20.6)	14.3 (7.8–23.6)	14.6 (8.0-25.2)	23.0 (12.1–65.5)	< 0.001
PPC	378 (40.2)	58 (30.9)	60 (31.9)	72 (38.3)	83 (44.1)	105 (55.9)	< 0.001
AKI	15 (1.6)	2 (1.1)	3 (1.6)	3 (1.6)	2 (1.1)	5 (2.7)	0.73
Hepatic dysfunction	16 (1.7)	3 (1.6)	0	2 (1.1)	2 (1.1)	9 (4.8)	0.005
Surgical site infection	27 (2.9)	4 (2.1)	7 (3.7)	4 (2.1)	3 (1.6)	9 (4.8)	0.31
Thrombus formation	15 (1.6)	3 (1.6)	2 (1.1)	3 (1.6)	3 (1.6)	4 (2.1)	0.95
Postoperative hypotension required vasopressors	70 (7.4)	9 (4.8)	4 (2.1)	11 (5.9)	15 (8.0)	31 (16.5)	< 0.001
Postoperative transfusion	348 (37.0)	39 (20.7)	34 (18.1)	59 (31.4)	86 (45.7)	130 (69.1)	< 0.001
Postoperative acidosis	339 (36.1)	45 (23.9)	52 (27.7)	63 (33.5)	83 (44.1)	96 (51.1)	< 0.001
Postoperative Hb	135.0 (113.8–158.0)	144.0 (125.0–166.0)	140.0 (118.8–160.0)	137.5 (116.0–164.0)	131.0 (112.0–155.0)	118.0 (102.0–142.0)	< 0.001
Postoperative HCT	41.4 (34.9–48.3)	44.1 (38.3–50.9)	43.0 (36.4–49.0)	42.1 (35.8–49.6)	40.3 (34.6–47.4)	36.5 (31.5–43.6)	< 0.001
Postoperative lactic acid	1.5 (1.0–2.3)	1.5 (1.0–2.2)	1.4 (1.0–2.0)	1.4 (1.2–2.1)	1.5 (1.0–2.3)	1.8 (1.2–2.9)	< 0.001
Unplanned reintubation	18 (1.9)	1 (0.5)	1 (0.5)	4 (2.1)	5 (2.7)	7 (3.7)	0.10

Data are presented as median (quartile) or *n* (%)

*AKI* acute kidney injury, *Hb* hemoglobin, *HCT* hematocrit, *ICU* intensive care unit, *LOS* length of stay, *PPC* postoperative pulmonary complication

### Secondary outcomes

The complications in different categories were as follows: postoperative pulmonary complications: 378 (40.2%), AKI 15 (1.6%), hepatic dysfunction: 16 (1.7%), surgical site infection: 27 (2.9%), thrombus formation: 15 (1.6%), and postoperative hypotension required vasopressors: 70 (7.4%) (Table 2).

The median (IQR) postoperative mechanical ventilation time was 14.3 (7.7–26.6) hours (Table 2). Higher fluid load was also associated with a higher risk of mechanical ventilation time ≥ 24 h (Q5: OR 2.212, 95% CI 1.101–4.445, *P* = 0.026, *P* for trend = 0.020) (Fig. 2).

Higher intraoperative fluid administration was associated with increased PPCs (*P* < 0.001), hepatic dysfunction (*P* = 0.005), postoperative hypotension required vasopressors (*P* < 0.001), acidosis (*P* < 0.001), blood transfusion (*P* < 0.001), lactic acid (*P* < 0.001), decreased hemoglobin (*P* < 0.001), and hematocrit (*P* < 0.001) (Table 2). However, these postoperative outcomes did not show statistically significant association with fluid load after adjusted with risk factors (Fig. 2).

## Discussion

In the current study, our data demonstrated that high fluid administration was associated with an increased risk of prolonged postoperative length of hospital stay and overall complication. Conversely, low fluid load (Q1) showed better postoperative outcomes. This study contributes to the existing literature by providing insights into the association between intraoperative fluid management and clinical outcomes in neonates.

Previous studies showed that a U-shaped relationship exists between perioperative fluid load and complications in non-cardiac surgery, indicating that both very high and very low fluid load can be harmful [1, 13, 23]. However, those studies were only conducted in adults, and there is a lack of research specifically focusing on neonates. It is important to consider that there may be differences in organ function between adults and neonates. In neonates, the effective renal blood flow and glomerular filtration rate (GFR) is increased with gestational age. After birth, the GFR increases significantly and reaches to adult levels by 2 years of age [19]. Due to the low GFR and renal blood flow in infants, especially in preterm infants, they have limited capacity to cope large volumes of fluids and are vulnerable to high fluid overload. It has been reported that early fluid accumulation is associated with increased mortality in critically ill children in the ICU [24]. Furthermore, fluid balance is associated with mechanical ventilation on postnatal day 14 in the extremely premature neonates [25]. This may explain why our study findings suggest a correlation between lower fluid infusion and better prognosis in neonates.

Recent studies highlighted the importance of ICU length of stay as a key measure of postoperative recovery. It is considered to be a proxy for acute physical recovery and serves as a significant predictor of long-term recovery [6]. In our study, we aimed to compare the association between different fluid load and prolonged LOS/complications to assess their impact on patient outcomes. Our findings revealed a positive correlation between the amount of fluid administration and the increase of prolonged LOS. Specifically, in patients who received higher fluid load (Q5), both prolonged LOS and complications were significantly increased. These results are consistent with previous research findings [1] and are similar to what has been found in the patients undergoing colon and rectal surgery [13, 26]. It is important to note that complications resulting from fluid overload may take time to resolve, leading to longer ICU stays. On the other hand, the lower fluid load (Q1) showed shorter postoperative LOS, suggesting that this may be a relatively suitable fluid regimen for neonates. However, further multicenter study is needed to provide additional evidence in supporting our finding.

Previous studies in adult patients showed that both very high and very low fluid loads are associated with an increased risk of mortality [1, 23]. In our study, we had a large cohort of neonates with varying admission types (emergency or elective), and surgery types. This allowed us to reduce bias related to fluid quantities and types and to objectively evaluate the influence of intraoperative fluids on outcomes. However, we did not find a significant association between fluid load and 30-day mortality risk after adjusted with various related factors although the high fluid load (Q5) was associated with a low rate of survival. In children admitted to pediatric ICU, a previous study demonstrated that there was a 6% increase in odds of mortality for every 1% increase in percentage fluid overload [27]. Further research is needed to further our understanding to the underlying mechanisms for this discrepancy and to investigate other potential factors that may contribute to the relationship between fluid load and mortality in neonates.

Excessive fluid administration can have harmful effects on the lungs, leading to pulmonary edema, acute respiratory distress syndrome (ARDS), and pneumonia. Pulmonary edema impairs gas exchange, increasing the risk of infection, respiratory failure, and the need for reintubation. Postoperative pulmonary complications are the most common surgical related complications and are known to significantly increase postoperative LOS, mortality, and healthcare costs. A meta-analysis of several trials demonstrated that higher intraoperative fluid load were associated with increased odds of postoperative pulmonary edema and pneumonia [28]. In our study, after adjusted with other factors, we did not find that the high intraoperative fluid load (Q5) was associated with a high risk of postoperative pulmonary complications. However, we did find a positive association between fluid load and the incidence of postoperative pulmonary complications and the duration of postoperative mechanical ventilation (≥ 24 h). This finding is consistent with a previous study showing that for every 10% increase in peak positive fluid balance, there is a 103% increase in the odds of requiring mechanical ventilation at postnatal day 14 [25]. Additionally, the positive fluid balance in the first postnatal week was linked to mechanical ventilation at postnatal day 7 in neonates born less than 36 weeks gestational age [29].

Previous studies reported that moderate fluid load has been shown to have the lowest risk of developing AKI [1, 23, 30]. Hypovolemia can lead to renal hypoperfusion, which can result in acute tubular necrosis and renal dysfunction [31]. On the other hand, hypervolemia reduced glomerular filtration and promoted renal parenchymal edema by increasing central venous pressure (CVP) [1]. This can affect renal uptake and lead to the accumulation of serum creatinine, ultimately leading to the occurrence of AKI [31]. In our study, we did not find a significant association between fluid load and AKI although AKI is common in critically ill neonates [4]. Further research with a larger sample size is needed to better understand the association between intraoperative fluid load and AKI in neonates.

This study has several limitations. First, the data were retrospectively collected from a single center, which may introduce regional limitations and potential biases. Secondly, our study only focused on short-term outcomes related to intraoperative fluid load, and the long-term outcomes are unknown. Thirdly, major surgery in general needs long surgery time and causes more traumatic injury to young patients. However, how much surgical related factors and surgical disease severity contributed to the findings of the current study is unknown. Future multicenter cohort studies are needed to determine the optimal fluid management strategies in neonates.

In conclusion, our study highlights the association between excessive fluid load and poor postoperative outcomes in neonates undergoing non-cardiac surgery. Neonates are sensitive and vulnerable, and even small treatment alterations can have a significant impact on outcomes. Therefore, identifying appropriate intraoperative fluid management strategies are crucial to minimize complications and promote faster postoperative recovery in this patient population.

## Data Availability

Available upon request to the corresponding author.

## Abbreviations

AKI: Acute kidney injury
CI: Confidence intervals
GFR: Glomerular filtration rate
ICU: Intensive care unit
IQR: Interquartile range
LOS: Length of hospital stay
OR: Odds ratios
PPCs: Postoperative pulmonary complications
ROC: Receiver operating characteristic
